# Comparison of the Phenolic Compound Profile and Antioxidant Potential of *Achillea atrata* L. and *Achillea millefolium* L.

**DOI:** 10.3390/molecules26061530

**Published:** 2021-03-11

**Authors:** Lysanne Salomon, Peter Lorenz, Marek Bunse, Otmar Spring, Florian C. Stintzing, Dietmar R. Kammerer

**Affiliations:** 1 WALA Heilmittel GmbH, Department of Analytical Development & Research, Section Phytochemical Research, 73087 Bad Boll, Germany; Lysanne.Salomon@wala.de (L.S.); Peter.Lorenz@wala.de (P.L.); Marek.Bunse@wala.de (M.B.); Florian.Stintzing@wala.de (F.C.S.); 2 Institute of Botany, Hohenheim University, 70599 Stuttgart, Germany; o.spring@uni-hohenheim.de

**Keywords:** *Achillea atrata* L., *Achillea millefolium* L., antioxidant activity, DPPH, phenolic metabolome

## Abstract

In the present study, *Achillea atrata* L. and *A. millefolium* L. were compared for the first time with regard to their phenolic compound profile and antioxidant activity by applying the 2,2-diphenyl-picryl hydrazyl radical assay. For this purpose, aerial plant parts were consecutively extracted with solvents of increasing polarity (dichloromethane, *n*-butanol, ethyl acetate), revealing that the *A. atrata* ethyl acetate fraction showed the highest antioxidant activity with an IC_50_ value of 12.2 ± 0.29 µg/mL compared to 17.0 ± 0.26 µg/mL for *A. millefolium*. Both species revealed the presence of luteolin, apigenin, centaureidin, and nevadensin exclusively in this most polar fraction, which are known as effective 2,2-diphenyl-picryl hydrazyl radical scavengers. The antioxidant capacity of the aforementioned fractions strikingly correlated with their total phenolic contents, which was highest in the ethyl acetate fraction of *A. atrata*. Characterization of the metabolite profiles of both *Achillea* species showed only marginal differences in the presence of key compounds, whereas the concentrations of individual compounds appeared to be species-specific. Our results suggest that *A. atrata*, based on its compound pattern and bioactivity characteristics, has similar qualities for phytotherapy as *A. millefolium*.

## 1. Introduction

Plants are sessile organisms and are exposed to a wide variety of different abiotic stress factors in constantly changing environments. Water deficiency, contamination of the soil with heavy metals, salinity, nutrient surplus or deficiency, high and low temperatures, extreme light, and UV-B radiation are only some of such abiotic stress factors that affect plants and strongly influence their growth and development [1,2,3]. Abiotic stress promotes the production of damaging reactive oxygen species (ROS) and nitrogen species within cells and leads to rapid changes in cellular redox homeostasis, resulting in peroxidation and destabilization of cellular membranes [1,2,3]. The accumulation of secondary metabolites in plant tissues such as phenolics is a typical adaptive response of plants to these adverse environmental conditions [1]. Plant phenolics are aromatic compounds with one or more hydroxyl groups and are biosynthesized in plants from phenylalanine and shikimic acid through the shikimic acid pathway [1,4,5,6]. When a plant is exposed to abiotic stress, the activity of phenylalanine ammonia lyase (PAL) and other enzymes necessary for phenolic biosynthesis is upregulated, resulting in increased phenol production to ensure plant survival and increase stress tolerance [1,5]. These antioxidant and radical scavenging properties of phenolics are crucial for the plant. Consequently, they are also attracting increasing interest in the preservation of human health and in preventing physiopathological conditions where oxidative damage is a hallmark [7,8,9]. Medicinal plants with a high level of these bioactive compounds play an important role in the prevention of chronic diseases, slowing down aging processes as well as reducing the risk of cardiovascular and neurodegenerative diseases [9,10]. Due to their health benefits, the search for novel sources of natural antioxidants for pharmaceutical and medicinal purposes is of growing interest.

In particular, the genus *Achillea*, consisting of more than 140 perennial species native to the Northern Hemisphere, is characterized by a pronounced antioxidant activity [11]. More than twenty *Achillea* species and subspecies including *A. millefolium*, which have been used as medicinal plants, have previously been assessed with regard to their anti-radical scavenging properties by investigating various extracts recovered with solvents of different polarities [11,12,13]. However, investigations into the antioxidant activity of the alpine species *A. atrata* have not been reported yet. Consequently, the aim of the present study was a first in-depth investigation of the radical scavenging capacity of *A. atrata* applying the 2,2-diphenyl-picryl hydrazyl (DPPH) radical in vitro assay as a model test system, which should form a basis for further assessment of the antioxidant potential of *A. atrata*, both in vitro and in vivo. Furthermore, the characterization of secondary metabolites with particular focus on phenolic compounds and the comparison of the compound profile and bioactivity with *A. millefolium* obtained from the same habitat should be performed to broaden our knowledge of *Achillea* species potentially applicable to pharmaceutical purposes.

## 2. Results

### 2.1. Phytochemical Comparison of A. atrata and A. millefolium

**Figure 1:**
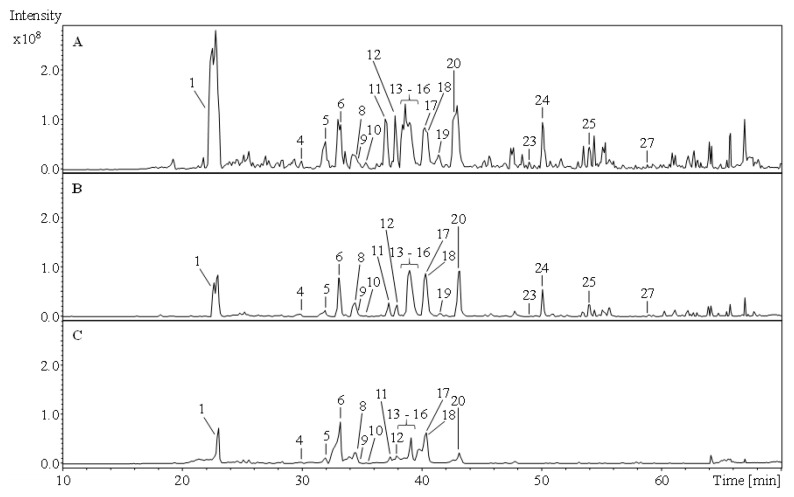
Comparison of total ion chromatograms (TIC) of the *Achillea atrata* acetone/water extract (**A**) and the corresponding ethyl acetate (**B**) and *n*-butanol (**C**) fractions. 1: chlorogenic acid; 4: quercetin-*O*-hexoside I; 5: 4-methyl-3-methoxy-9a-hydroxyligballinol-*O*-glucoside (formate adduct); 6: quercetin-3-*O*-rutinoside; 8: luteolin-hexoside; 9: quercetin-*O*-hexoside II; 10: mearnsetin-hexoside; 11: isorhamnetin-*O*-hexoside I; 12: kaempferol-3-*O*-rutinoside II; 13–16: dicaffeoylquinic acid I–VI; 17: apigenin-7-*O*-glucoside; 18: isorhamnetin-*O*-hexoside II; 19: dicaffeoylquinic acid V; 20: dicaffeoylquinic acid VI; 23: caffeoyl-feruloylquinic acid; 24: luteolin; 25: apigenin; 27: nevadensin.

**Figure 2:**
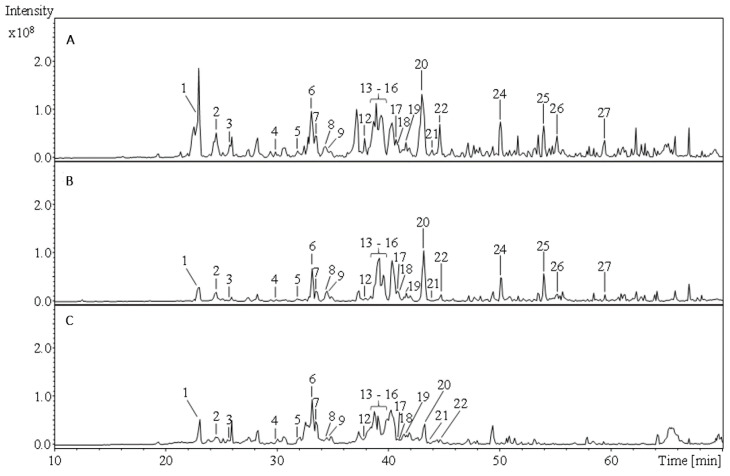
Comparison of total ion chromatograms (TIC) of the *Achillea millefolium* acetone/water extract (**A**) and the *Achillea millefolium* ethyl acetate (**B**) and *n*-butanol (**C**) fractions. 1: chlorogenic acid; 2: *p*-coumaroyl acid derivative; 3: apigenin-6,8-di-*C*-hexoside; 4: quercetin-*O*-hexoside I; 5: 4-methyl-3-methoxy-9a-hydroxyligballinol-*O*-glucoside (formate adduct); 6: quercetin-3-*O*-rutinoside; 7: kaempferol-3-*O*-rutinoside I; 8: luteolin-hexoside; 9: quercetin-hexoside II; 10: mearnsetin-hexoside; 11: isorhamnetin-*O*-hexoside I; 12: kaempferol-3-*O*-rutinoside II; 13–16: dicaffeoylquinic acid I–VI; 17: apigenin-7-*O*-glucoside; 18: isorhamnetin-*O*-hexoside II; 19: dicaffeoylquinic acid V; 20: dicaffeoylquinic acid VI; 21: dicaffeoylquinic acid VII; 22: cinnamic acid derivative; 23: caffeoyl-feruloylquinic acid; 24: luteolin; 25: apigenin; 26: centaureidin; 27: nevadensin.

Both *Achillea* species were fractionated with solvents of different polarity (i.e., with dichloromethane, acetone/water, ethyl acetate, and *n*-butanol). Potential correlations between secondary metabolites and antioxidant activity, but also species-specific metabolites should be identified. The phenolic compounds of the polar acetone/water extracts and of the ethyl acetate and *n*-butanol fractions were characterized based on their UV characteristics, HPLC retention times, specific mass spectra, and comparison with reference substances or literature data (Table 1, Figure 1 and Figure 2). Individual compounds of the non-polar dichloromethane fractions were analyzed using gas chromatography-mass spectrometry (GC-MS) and assigned based on their specific mass spectrometric data as well as retention times in comparison with the NIST MS database (Table 2).

A total of 27 phenolic compounds were identified, among which 21 were detected in the acetone/water extract of *A. atrata* and 23 in the corresponding extract of *A. millefolium*. By comparing the compound profiles of both *Achillea* species, nine differences were detected: the *p*-coumaroyl acid derivative (2), apigenin-6-8-di-*C*-hexoside (3), kaempferol-3-*O*-rutinoside I (7), dicaffeoylquinic acid VII (21), the cinnamic acid derivative (22), and centaureidin (26) could only be assigned for *A. millefolium*. In contrast, the occurrence of mearnsetin-hexoside (10) and of isorhamnetin-*O*-hexoside I and II (11, 18) was specific for *A. atrata*. The remaining compounds (1, 4–6, 8, 9, 12–25, 27) were detected in both *Achillea* species, with the main components being caffeic acid derivatives, kaempferol-, luteolin-, quercetin-, and apigenin-glycosides. All compounds characterized in the acetone/water extracts were also detected in the respective ethyl acetate fractions. Marked differences between the ethyl acetate and the *n*-butanol fractions were found in the occurrence of luteolin, apigenin, centaureidin, and nevadensin exclusively in the ethyl acetate fraction. GC-MS analyses of the dichloromethane fractions revealed a total of 23 constituents. However, only 10 of the latter were detected in the *A. atrata* dichloromethane fraction including the monoterpenes α-thujene (1), γ-terpinene (5), the sesquiterpenes caryophyllene (15), caryophyllene oxide (17), and α-eudesmol (18) as well as the alkanes I–V (19–23).

### 2.2. Antioxidant Capacity and Contents of Phenolics and Volatile Compounds

The radical scavenging capacity of the aforementioned extracts and fractions when applying the DPPH assay is reported in Table 3 by specifying the respective IC_50_ values, thus allowing direct comparison. In general, the dichloromethane fractions did not reveal a radical scavenging potential, even at higher concentrations (750 µg/mL). The ethyl acetate fractions of both *Achillea* species showed the highest activity compared to the respective *n*-butanol fractions. Comparative analyses of the two species revealed the ethyl acetate fraction of *A. atrata* to exhibit both the highest antiradical potential with an IC_50_ value of 12.2 ± 0.3 µg/mL and the highest phenolic content of 250 ± 2.5 mg GAE/g dry weight (Table 4). However, the activity of all *Achillea* fractions examined was inferior to the reference compound Trolox with an IC_50_ value of 7.5 ± 0.1 µg/mL.

## 3. Discussion

Medicinal plants with their unmatched chemical diversity provide unlimited opportunities for the discovery of novel plant-based medicinal products [24]. According to the World Health Organization (WHO), more than 80% of the world’s population still rely on plant-based traditional medicines for primary health care [14,15]. However, only for a small proportion of ethnopharmaceutically applied medicinal plants have bioactivity studies been performed so far. As an example, among the ~250,000 higher plant species worldwide, only about 5 to 10% have been analyzed with regard to their constituents and associated bioactivity [31]. The utilization of novel natural antioxidants from medicinal plants, which might help to mitigate oxidative damage by protecting lipids from oxidation and thus might also be beneficial to human health, is of particular interest [11]. In order to meet the high demand for herbal medicines and to provide evidence for documented ethnobotanical applications of medicinal plants, the identification of further potential health-promoting plant species and the exploration of correlations between chemical composition and bioactivity is of utmost importance. To the best of our knowledge, only three studies on the bioactivity of *A. atrata*, also considering the identification of individual components, have been reported so far [32,33,34]. In contrast to these investigations, our studies report for the first time the characterization of the phenolic compound profile and antioxidant activity of the alpine species *A. atrata*, together with a comparison of the respective traits of *A. millefolium* originating from the same habitat, to exclude any impact of edaphic and climatic factors.

The highest anti-radical capacity of the extracts was found in the ethyl acetate fractions of both species. The phytochemical comparison of these two *Achillea* species confirmed the findings of our previous study [34], indicating the differences in the occurrence of major phenolic components of aerial parts to be marginal. In contrast, the amounts of individual phenolic compounds appear to be species-specific. Furthermore, the results presented here confirm previous studies indicating that extraction with polar organic solvents such as ethyl acetate results in highest phenolic yields, which goes along with the pronounced antiradical scavenging potential of such extracts [11,13,35]. Upon direct comparison of different *Achillea* species under similar extraction conditions, the following ranking of their IC_50_ value can be established according to the literature data: *A. aucherii* (IC_50_ = 844 µg/mL) [10] > *A. kellalensis* (IC_50_ = 518 µg/mL) [10] > *A. pachycephalla* (IC_50_ = 248 µg/mL [10] > *A. biebersteinii* Afan. (IC_50_ = 89.90 µg/mL) [28] > *A. millefolium* (IC_50_ = 16.95 µg/mL) [our results] > *A. atrata* (IC_50_ = 12.19 µg/mL) [our results] > *A. moschata* (IC_50_ = 3.18 µg/mL) [13]. Only the very closely related alpine species *A. moschata* had a higher antioxidant capacity than *A. atrata*. This might be due to the fact that in this study, the aerial parts of *A. moschata* were collected from higher altitudes of 2400 m a.s.l. (Rhaetian Alps, Italy) [13], whereas our investigated *A. atrata* plants were harvested at an altitude of 2100 m a.s.l. (Nufenen Pass, Switzerland). Altitude might be a decisive factor for the accumulation of phenolics in plant tissues and an increased antioxidant potential to protect against the damaging influence of UV-B radiation, which increases with altitude [36]. On the other hand, the IC_50_ values of all other *Achillea* species were inferior to that of *A. atrata*, with the latter exhibiting a 43-fold higher activity than *A. kellalensis* extracts [13]. This high antioxidant activity of *A. atrata* is presumably attributed to the high content of phenolic compounds (250 ± 2.5 mg GAE/g dry weight) compared to *A. millefolium* (175 ± 1.0 mg GAE/g dry weight). Regardless of the species, the fractionation and subsequent phytochemical characterization revealed that luteolin, apigenin, nevadensin, and centaureidin were detected exclusively in the corresponding ethyl acetate fractions. A correlation between the contents of these phenolic compounds and an increased antimicrobial activity has already been demonstrated in our previous study [34]. It is also conceivable that an increased antioxidant capacity goes along with the occurrence of these four compounds, which are known as effective DPPH radical scavengers [37,38,39]. Future studies are required, aiming at the identification of further antioxidant components in the most potent ethyl acetate fraction of *A. atrata*. Moreover, the DPPH assay is a widely used method for determining antioxidant activity, not only of *Achillea* extracts [13,28,40], thus allowing direct comparison with our results, but also of other plant extracts. Nevertheless, further in vitro assays should be performed to confirm our first insights into the high antioxidant potential of *A. atrata* and overcome the limitations of the DPPH assay.

## 4. Conclusions

This study reported, for the first time, the pronounced antiradical activity of *A. atrata* together with its high phenolic contents compared to *A. millefolium*. Consequently, *A. atrata* with its high antioxidant potential might be an alternative source of natural antioxidants in pharmaceutical and medicinal applications in the future. In particular, alpine plant species characterized by high contents of phenolic compounds, which are accumulated in plant tissues in response to damaging environmental conditions such as UV-B radiation, might be used as potential future phytopharmaceuticals, being promising alternatives to synthetic active substances.

## 5. Materials and Methods

### 5.1. Plant Material

Both *Achillea* species analyzed in the present study (i.e., *A. atrata* and *A. millefolium*) were collected at the flowering stage in July 2019, on the Nufenen Pass (Switzerland) at an altitude of 2100 m a.s.l. Aerial parts of the plants were air-dried at room temperature and subsequently stored in paper bags until analysis. Both *Achillea* species were identified by Dr. phil. Rhinaixa Duque-Thüs (Institute of Botany, Hohenheim University, Stuttgart, Germany), and voucher specimens were deposited in the herbarium of the Institute of Botany at Hohenheim University (*A. atrata*: HOH-022704; *A. millefolium*: HOH-022706).

### 5.2. Chemicals

Dichloromethane, acetone, ethyl acetate, and *n*-butanol for plant material extraction were purchased from Merck KGaA (Darmstadt, Germany). Acetonitrile (LC-MS grade), formic acid (98%), and methanol (LC-MS grade) for LC-MS^n^ analyses were obtained from Sigma–Aldrich (Steinheim, Germany). Purified water (0.056 μS/cm) from a Purelab Option-Q system (Elga Berkefeld GmbH, Celle, Germany) was used throughout. The DPPH photometric assay and the quantitation of total phenolic compounds were performed with 2,2-diphenyl-1-picryl hydrazyl radical (DPPH), Folin–Ciocalteu’s phenol reagent, and sodium carbonate from Th. Geyer GmbH & Co. KG (Renningen, Germany). The following reference substances were used: luteolin and 5-caffeoylquinic acid (chlorogenic acid) from PhytoLab GmbH & Co. KG (Vestenbergsgreuth, Germany); quercetin-3-*O*-rutinoside, apigenin, and apigenin-7-*O*-glucoside from Carl Roth GmbH & Co. KG (Karlsruhe, Germany); gallic acid monohydrate from Sigma–Aldrich (Steinheim, Germany); and Trolox from Cayman Chemical (Ann Arbor MI, USA).

### 5.3. Extraction and Fractionation of the Plant Material

Fractionation was performed by applying the DPPH spectrophotometric assay and aimed at the identification of those substances from the complex natural compound mixture of *A. atrata* and *A. millefolium*, which are responsible for antioxidant activity. For this purpose, air-dried aerial parts of the plants (30 g each) were pulverized with a mortar and pestle for 15 min. Subsequently, the plant material was defatted with 800 mL dichloromethane. To prevent oxidation during extraction, the suspension was bubbled with nitrogen for 15 min. After incubation for 24 h at 4 °C under light exclusion, the suspensions were filtered over Celite^®^ through a Büchner funnel by vacuum suction. This fraction was further processed for volatile compounds analysis. For this purpose, dichloromethane was removed by rotary evaporation to yield 0.44 g of *A. millefolium* and 0.94 g of *A. atrata* extract, respectively. Subsequently, the solid residues were extracted twice with 800 mL acetone/water (8:2; *v/v*) for 24 h at 4 °C in the dark. The suspensions were again filtered over Celite^®^ through a Büchner funnel by vacuum suction. Acetone was removed from the filtrates in vacuo by rotovaporation at 34 °C. The resulting aqueous phases were subsequently extracted with ethyl acetate (2 × 200 mL) and *n*-butanol (2 × 200 mL). The respective phases were evaporated to dryness to yield 0.71 g residue of the ethyl acetate extract from each species as well as 0.37 g (*A. atrata*) and 0.71 g (*A. millefolium*) residue of *n*-butanol extract [34,41]. In parallel to this consecutive extraction with different solvents, 4 g of comminuted *A. atrata* and *A. millefolium* plant material were extracted twice with 80 mL acetone/water (8:2; *v/v*) under exclusion of light for 24 h to obtain a total compound extract. The suspensions were filtered as above-mentioned and evaporated to dryness, resulting in crude extract yields of 0.45 g for *A. atrata* and 0.45 g for *A. millefolium*, respectively.

### 5.4. LC-MS^n^ Analyses for Phenolic Compound Characterization

The chromatographic separation and identification of phenolic compounds were performed with an Agilent 1200 HPLC (Agilent, Waldbronn, Germany) system connected with an HCT ultra ion trap MS detector interfaced with an electrospray ionization (ESI) ion source (Bruker Daltonik, Bremen, Germany). A binary gradient elution system described previously was applied and consisted of 0.1% formic acid (*v/v*; eluent A) and acetonitrile (eluent B) [41]. Chromatographic separation was performed on a Kinetex^®^ C18 reversed-phase column (2.6 μm particle size, 150 × 2.1 mm i.d., Phenomenex Ltd., Aschaffenburg, Germany) at a flow rate of 0.21 mL/min. UV absorption of the column eluates was recorded at 210, 254, 280, and 366 nm. All experiments were performed in triplicate. The software Agilent Chemstation (Rev. B.01.03 SR1) (Agilent, Waldbronn, Germany) and Bruker Daltonik esquire control (Version 6.1) (Bruker Daltonik GmbH, Bremen, Germany) were used for data acquisition and processing [34,41].

### 5.5. Gas Chromatography-Mass Spectrometry (GC-MS) Analyses for Volatile Compound Assessment

GC/MS analyses were carried out using a PerkinElmer Clarus 500 gas chromatograph (PerkinElmer Inc., Waltham, MA, USA) equipped with split injection (split ratio: 30:1, injection volume: 1.0 μL) coupled to a mass spectrometer. The column used was a Zebron ZB-5ms capillary column (60 m × 0.25 mm inner diameter × 0.25 μm film thickness, 5% phenylpolysiloxane, and 95% dimethylpolysiloxane coating; Phenomenex, Torrance, CA, USA). Helium with a flow rate of 1 mL/min was the carrier gas. The column oven temperature program was 100–320 °C at 4 °C per min with a final hold time of 30 min. The mass spectrometer was run in electron ionization (EI) mode and set at 70 eV. The software Turbomass version 5.4.2 (PerkinElmer Inc., Waltham, MA, USA) was used for data acquisition and processing. All experiments were carried out in triplicate. Individual compounds of the dichloromethane fraction of *A. atrata* and *A. millefolium* were assigned based on their specific MS data as well as retention times in comparison with the NIST MS database (NIST Mass Spectral Library, NIST2011, V 2.0, PerkinElmer Inc., Waltham, MA, USA) and reference compounds [34,41].

### 5.6. 2,2-Diphenyl-Picryl Hydrazyl (DPPH) Spectrophotometric Assay for Assessing Radical Scavenging Capacity

The 2,2-diphenyl-picryl hydrazyl (DPPH) radical scavenging capacity of the *Achillea* fractions was determined according to a protocol of Heinrich et al. [42] with some modifications. In brief, 200 µL aliquots of each fraction and of the reference antioxidant Trolox, at five different concentrations for each extract (ethyl acetate: 2.5–20 µg/mL; *n*-butanol: 20–100 µg/mL; dichloromethane: 50–750 µg/mL; Trolox: 1.5–12.5 µg/mL) were added to 1800 µL of DPPH solution (100 µM) and incubated at 37 °C in the dark. After a reaction time of 30 min, the decrease in absorbance was measured at 516 nm, using a UV/VIS spectrophotometer (Lambda 35, Perkin Elmer, Rodgau-Juedesheim, Germany). The experiments were performed in triplicate. IC_50_ values, indicating a 50% decrease of DPPH solution absorbance referred to the blank, were calculated by plotting absorbance at 516 nm against the corresponding concentrations and subsequent regression analyses.

### 5.7. Folin–Ciocalteu Method for Total Phenolics Quantitation

The amount of total phenolic compounds in the *Achillea* extracts and fractions was determined by the Folin–Ciocalteu method using the protocol of Giorgi et al. [9]. Gallic acid was used as a reference substance. The quantitation was performed on the basis of the gallic acid standard calibration curve, and the results were reported as mg gallic acid equivalents (GAE) per g dry weight. All experiments were carried out in triplicate.

## Figures and Tables

**Table 1:** Spectroscopic data (UV, mass spectrometry (MS)) and high performance liquid chromatography (HPLC) retention times (Rt) of secondary metabolites of *A. atrata* and *A. millefolium* [acetone/water extracts (aw), ethyl acetate fractions (EtOAc) and *n*-butanol fractions (*n*-but)]. Only the most intense *m/z* ratios of the collision-induced dissociation (CID) experiments are illustrated.

Peak No.	Rt [min]	Peak Assignment	UV λmax [nm]	MS^n^ Data [*m/z*]			*A. atrata*			*A. millefolium*			Ref.
				MS ^1^	MS ^2^	MS ^3^	aw	EtOAc	*n*-but	aw	EtOAc	*n*-but	
1	22.6 23.0	Chlorogenic acid	216, 326	353	191	173	+	+	+				RS*^1^/[14]*^2^
										+	+	+	
2	24.5	*p*-Coumaroyl acid derivative	326	387	207	163	-	-	-	+	+	+	[15] *^1^
3	25.5	Apigenin-6,8-di-*C*-hexoside	194, 280	593	473	353	-	-	-	+	+	+	[16] *^1^/[17] *^2^
4	30.0	Quercetin-*O*-hexoside I	282, 342	463	301	283	+	+	+	+	+	+	[15] *^1^/[18] *^2^
5	31.8 32.1	4-Methyl-3-methoxy-9a- hydroxyligballinol-*O*-glucoside	202, 278 202, 276	565	339	324				+	+	+	[16] *^1^
							+	+	+				
6	33.0	Quercetin-3-*O*-rutinoside	200, 374	609	301	179	+	+	+	+	+	+	RS *^1^/[19] *^2^
7	33.4	Kaempferol-3-*O*-rutinoside I	202, 342	593	285	255	-	-	-	+	+	+	[16] *^1^/[20] *^2^
8	34.4	Luteolin-hexoside	266, 348	447	285	255	+	+	+	+	+	+	[21] *^1^/[22] *^2^
9	34.4 34.7	Quercetin-*O*-hexoside II	204, 328	463	301	151				+	+	+	[15] *^1^/[18] *^2^
							+	+	+				
10	35.3	Mearnsetin-hexoside	198, 336	493	331	316	+	+	-	-	-	-	[23] *^1^/[24] *^2^
11	36.5	Isorhamnetin-*O*-hexoside I	204, 328	477	315	300	+	+	+	-	-	-	[16] *^1^/[18] *^2^
12	37.9 38.1	Kaempferol-3-*O*-rutinoside II	266, 342	593	285	255	+	+	+				[16] *^1^/[20] *^2^
										+	+		
13	38.1	Dicaffeoylquinic acid I	204, 324	515	353	191	+	+	+	+	+	+	[16] *^1^/[12] *^2^
14	38.4	Dicaffeoylquinic acid II	204, 324	515	353	191	+	+	+	+	+	+	[16] *^1^/[12] *^2^
15	38.6	Dicaffeoylquinic acid III	204, 324	515	353	191	+	+	+	+	+	+	[16] *^1^/[12] *^2^
16	39.3 39.9	Dicaffeoylquinic acid IV	204, 324	515	353	191				+	+	+	[16] *^1^/[12] *^2^
							+	+	+				
17	40.2	Apigenin-7-*O*-glucoside	268, 338	431	269	225	+	+	+	+	+	+	RS *^1^/[17] *^2^
18	40.6	Isorhamnetin-*O*-hexoside II	200, 332	447	315	300	+	+	+	-	-	-	[16] *^1^/[18] *^2^
19	41.4	Dicaffeoylquinic acid V	194, 324	515	353	191	+	+	-	+	+	+	[16] *^1^/[12] *^2^
20	43.0	Dicaffeoylquinic acid VI	216, 326	515	353	191	+	+	+	+	+	+	[16] *^1^/[12] *^2^
21	43.8	Dicaffeoylquinic acid VII	216, 326	515	353	191	-	-	-	+	+	+	[16] *^1^/[12] *^2^
22	44.6	Cinnamic acid derivative	-	549	387	369	-	-	-	+	+	+	[23] *^1^
23	48.4	Caffeoyl-feruloylquinic acid	196, 326	529	367	191	+	+	-	-	-	-	[25] *^1^/[26] *^2^
24	50.1	Luteolin	252, 346	285	241	217	+	+	-	+	+	-	RS *^1^/[19] *^2^
25	54.0	Apigenin	286, 332	269	225	-	+	+	-	+	+	-	RS *^1^/[17] *^2^
26	55.9	Centaureidin	234, 314	359	344	329	-	-	-	+	+	-	[27] *^1^/[28] *^2^
27	57.9	Nevadensin	332	343	328	313	+	+	-	+	+	-	[29] *^1^/[30] *^2^

RS: Reference standard; + detected; - not detected; *^1^ Reference: LC-MS^n^ data; *^2^ Reference: Substance identification in *Achillea* spec.

**Table 2:** Gas chromatography-mass spectrometry (GC-MS) analyses of *A. atrata* and *A. millefolium* dichloromethane fractions (DCM).

Peak No.	Peak Assignment	R_t_ [min]	Calc. M_r_ [Da] (tms)	Characteristic Fragments, *m/z* (BPI [%])	*A. atrata*	*A. millefolium*
1	α-Thujene	6.29	136.15	93 (100), 92 (42), 91 (50), 79 (20), 77 (24), 63 (9)	+	+
2	Bornylene	6.57	136.15	93 (100), 89 (29), 84 (32), 79 (25), 72 (88), 63 (29)	-	+
3	β-Thujene	6.81	136.15	93 (100), 91 (43), 79 (26), 77 (36), 69 (9)	-	+
4	Sabinene	6.98	136.15	93 (100), 91 (32), 79 (19), 77 (17), 69 (21), 67 (8)	-	+
5	γ-Terpinene	7.34	136.15	93 (100), 92 (22), 91 (50), 77 (29), 57 (4)	+	-
6	Eucalyptol	7.86	154.14	111 (36), 108 (59), 84 (89), 81 (100), 71 (68), 67 (32), 55 (33)	-	+
7	β-Terpineol	8.53	154.14	93 (70), 92 (33), 84 (25), 71 (100), 64 (16), 55 (49)	-	+
8	α-Thujone	9.37	152.12	110 (58), 109 (27), 95 (41), 81 (100), 79 (18), 69 (49), 68 (57)	-	+
9	Borneol	9.61	154.14	95 (100), 77 (94), 74 (30), 72 (51), 69 (25), 65 (30), 57 (56)	-	+
10	Camphor	10.47	152.12	95 (100), 83 (23), 81 (63), 69 (27), 67 (17), 55 (20)	-	+
11	(+)-Borneol	11.03	154.14	110 (18), 95 (100), 67 (9)	-	+
12	α-Terpineol	11.48	154.14	136 (54), 121 (52), 93 (100), 89 (20), 81 (36), 77 (24), 59 (95)	-	+
13	β-Bisabolol	12.84	222.20	82 (100), 78 (26), 73 (31), 65 (19), 58 (18), 55 (21), 53 (36)	-	+
14	Isoborneol	13.92	154.14	95 (100), 89 (20), 79 (22), 77 (15), 70 (29), 68 (26), 64 (18)	-	+
15	Caryophyllene	17.14	204.19	105 (46), 93 (100), 91 (94), 81 (23), 79 (61), 77 (33), 55 (52)	+	+
16	(+)-Nerolidol	21.83	222.20	107 (39), 93 (100), 81 (39), 79 (22), 71 (43), 67 (36), 55 (31)	-	+
17	Caryophyllene oxide	22.95	220.18	107 (38), 106 (33), 95 (48), 93 (68), 91 (55), 79 (100), 69 (33)	+	+
18	α-Eudesmol	24.00	222.20	204 (96), 161 (100), 149 (40), 108 (32), 93 (48), 79 (27), 59 (79)	+	+
19	Alkane I	40.19	-	113 (7), 99 (90), 85 (83), 71 (94), 57 (100), 55 (22)	+	+
20	Alkane II	44.27	-	113 (10), 99 (20), 85 (76), 71 (87), 57 (100), 55 (19)	+	+
21	Alkane III	48.05	-	113 (12), 99 (30), 85 (84), 71 (97), 69 (19), 57 (100), 55 (22)	+	+
22	Alkane IV	51.57	-	207 (4), 99 (32), 97 (20), 85 (85), 71 (99), 57 (100), 55 (16)	+	+
23	Alkane V	54.88	-	209(6), 99 (28), 85 (83), 83 (25), 71 (99), 57 (100), 55 (16)	+	+

**Table 3:** Antioxidant capacity of *A. atrata* and *A. millefolium* (*n* = 3; ± SD).

Samples	Regression Equation	R^2^	IC_50_ [µg/mL] ± SD
Trolox	y = 6.5233x + 1.3342	0.9975	7.5 ± 0.1
***A. atrata***			
ethyl acetate fraction	y = 4.0888x − 0.1440	0.9983	12.2 ± 0.3
*n*-butanol fraction	y = 0.6263x + 2.3083	0.9984	76.15 ± 0.3
dichloromethane fraction	-	-	-
***A. millefolium***			
ethyl acetate fraction	y = 3.0799x − 2.2077	0.9964	17.0 ± 0.3
*n*-butanol fraction	y = 0.6131x − 0.4695	0.9988	82.3 ± 0.8
dichloromethane fraction	-	-	-

SD: Standard deviation.

**Table 4:** Total phenolic content of *A. atrata* and *A. millefolium* (*n* = 3; ± SD).

Samples	Phenolic Content (mg GAE/g DW ± SD)
***A. atrata***	
ethyl acetate fraction	250 ± 2.5
*n*-butanol fraction	70 ± 1.0
dichloromethane fraction	-
***A. millefolium***	
ethyl acetate fraction	175 ± 1.0
*n*-butanol fraction	80 ± 1.5
dichloromethane fraction	-

GAE: Gallic acid equivalents; SD: Standard deviation; DW: Dry weight.

## Data Availability

The data presented in this study are available in manuscript.
